# Global distribution of white spot syndrome virus genotypes determined using a novel genotyping assay

**DOI:** 10.1007/s00705-019-04265-2

**Published:** 2019-05-27

**Authors:** J. Oakey, C. Smith, D. Underwood, M. Afsharnasab, V. Alday-Sanz, A. Dhar, S. Sivakumar, A. S. Sahul Hameed, K. Beattie, A. Crook

**Affiliations:** 1 Biosecurity Sciences Laboratory, Biosecurity Queensland, Queensland Department of Agriculture and Fisheries, 39 Kessels Road, Coopers Plains, QLD 4108 Australia; 2 Department of Aquatic Animal Health and Diseases, Iranian Fisheries Research Organization, Tehran, Iran; 3 National Aquaculture Group, King Abdul Aziz Rd, Al Murjan, Jeddah, 23715 Kingdom of Saudi Arabia; 4 School of Animal and Comparative Biomedical Sciences, The University of Arizona, 1041 E Lowell St, Tucson, AZ 85721 USA; 5 Aquatic Animal Health Laboratory, C. Abdul Hakeem College, Melvisharam, Vellore District, Tamil Nadu India; 6 Biosecurity Queensland, Queensland Department of Agriculture and Fisheries, 41 George Street, Brisbane, 4000 Australia

## Abstract

White spot disease, caused by infection with white spot syndrome virus (WSSV), is a serious panzootic affecting prawn aquaculture. The disease has spread rapidly around the prawn-culturing regions of the world through a number of previously identified mechanisms. The ability to distinguish and trace strains of WSSV is of great benefit to identify, and then limit, the translocation routes of the disease. Here, we describe a novel genotyping method using 34 short tandem repeat regions of the viral genome concurrently. This technique is highly sensitive to strain differences when compared to previous methods. The efficacy of the described method is demonstrated by testing WSSV isolates from around the globe, showing regional genotypic differences. The differences in the genotypes were used to create a global minimum spanning network, and in most cases the observed relationships were substantiated with verification of transboundary movement. This novel panel of STR markers will provide a valuable epidemiological tool for white spot disease. We have applied this to an outbreak of the disease in Queensland, Australia, that occurred in 2016. While the results indicate that the source of this outbreak currently remains cryptic, the analyses have provided valuable insights with which to further study the origins of the strains involved.

## Introduction

White spot disease (WSD) is a serious panzootic affecting prawn aquaculture. The disease is caused by white spot syndrome virus (WSSV), a large double-stranded circular DNA virus and currently the only member of the genus *Whispovirus* and family *Nimaviridae* [1]. In intensive aquaculture systems, mortality can be rapid (3-10 days) and occurs at a rate of up to 100% [2, 3]. The economic cost of the disease on the prawn aquaculture industry worldwide has been estimated at up to US$15 billion since the emergence and initial spread of the disease, increasing at a rate of US$1 billion annually, equating to approximately 10% of global prawn production [4].

The first reports of white spot disease in penaeids were in mainland China and Taiwan in 1992 [2, 5, 6]. By the end of the decade, the disease had spread to Korea [7], Japan [8, 9], and throughout South-East Asia (Vietnam, Thailand, Malaysia, Indonesia) and India [10, 11]. This rapid proliferation of the disease was most likely through transboundary movement of infected animals. In the 1990s the disease was reported also in United States of America [12] and by 1999 WSSV was detected in Central and South America. WSSV was found in wild prawns in retrospective analysis by *in situ* hybridisation of histology samples from Ecuador from 1996, prior to disease reported in 1999 [13]. In 2001, WSSV was reported also in prawn farms of Khuzestan on the northern Persian Gulf coast in Iran and over several other Iranian provinces over the next decade [14]. In 2010, WSSV was observed in Saudi Arabia, greatly affecting the *Penaeus indicus* industry until 2013, when the industry was replaced with specific-pathogen-free (SPF) and specific-pathogen-tolerant (SPT) *Penaeus* (*Litopenaeus*) *vannamei* and the disease was considered eradicated [15]. By 2012, WSSV was reported to be endemic in wild penaeids from the coast of Iraq [16].

In addition, there have been incursions of the disease to other prawn-farming regions of the world where containment and biosecurity measures have resulted in reports of eradication or subsequent low levels of sporadic disease, including Spain, Mozambique and Madagascar [11]. Transmission to wild crustaceans was observed in Darwin (Northern Territory, Australia) in 1999 following inadvertent feeding of imported prawns to crustaceans in a research facility that discharged water into Darwin Harbour. The harbour and surrounding waters were declared free of WSSV in 2000, and it was considered that the infection was at a sufficiently low level as to be unsustainable [17].

In November 2016, WSSV was identified following the onset of disease in a prawn farm near Brisbane, Queensland, Australia. Previously, white spot disease had not been diagnosed in Australian prawn farms, and Australia was considered to be free of the virus (despite the aforementioned Darwin incident). The disease showed rapid spread and high mortalities, affecting seven farms by February 2017. A low number of wild-caught crustaceans in the adjacent Logan River and in Moreton Bay also tested positive for the virus. In 2018, a large surveillance program of wild crustaceans in Moreton Bay detected considerable numbers of test-positive animals in the north of Moreton Bay, but not in the south near the mouth of the Logan River (K. Beattie, personal observation).

The prawn farming industry in Queensland is valued at approximately AU$87 million annually (http://www.daf.qld.gov.au), and the potential impact of establishment of endemic white spot disease would be severe. Hence, an important factor within the incursion investigation is the epidemiological analysis of the source, the patterns and the movement of the virus based upon strain identification and differentiation. The data are used to shape biosecurity decisions and inform risk analysis to help prevent future incursions of this and other exotic penaeid pathogens.

We recently published the whole genome sequence of WSSV-AU [18], the virus detected in a sample from the first Queensland property identified as infected with white spot disease. Analysis of the genome for genomic markers previously reported by Marks *et al.* (2004) [19] to show variation among WSSV strains was unable to associate the virus in South East Queensland with any previously reported genotype. The differing types of the loci hindered cumulative analysis or testing of high sample numbers, and the complexity of the markers limited their utility as a large-scale epidemiological tool. Although the scientific literature contains many reports from endemic regions with local studies using only one or a few of these markers, these were of limited epidemiological use, as many alleles were reportedly common to multiple regions. It was concluded that alternative markers were required for epidemiological tracing [18].

Examination of the WSSV-AU sequence aligned with other published WSSV genome sequences showed a number of variations in copy number of triplet-base motifs (short tandem repeats, STRs) in a similar way to microsatellite polymorphism. STRs have been used frequently to identify individuals, evolutionary processes, and kinships and for population/cluster analysis in eukaryotes [20], prokaryotes [21], and some of the larger viruses [22]. The high levels of polymorphism associated with STRs, the speed of processing, and the potential to simultaneously isolate and study large numbers of loci provide a capacity for detecting comparable differences among different levels of hierarchal clustering. Here, we describe the application of 34 STRs observed in WSSV to achieve a sensitive genotyping method. Furthermore, we demonstrate the utility of the genotyping technique to discriminate WSSV strains between, within and among the principal WSSV-affected regions of the world.

## Materials and methods

The alignment of the WSSV-AU sequence (MF768985) with Taiwanese (AF440570), Thai (AF369029), Chinese (AF332093) and Korean (JX515788) WSSV sequences was examined using Integrative Genome Viewer 2.3.98 [23, 24] to manually identify potential trimeric STR markers with variation in copy number in at least one of these reference sequences compared to WSSV-AU. Primers in the conserved sequence flanking these loci were designed using BatchPrimer3 [25], pre-selecting amplicon size less than 500 bp and with as much consistency in melting temperatures as possible among all primers. Notional size ranges for the loci were estimated up to a 30-base increase or decrease compared to the alleles observed in WSSV-AU, and hypothetical fragments were analysed in Multiplex Manager [26] to design a 4-dye multiplexed analysis protocol with as few reactions as possible while avoiding primer cross-reactivity or overlapping of fragments labelled with same dye, and using common primer annealing temperatures. Primers were redesigned as necessary to minimise the number of reactions needed. Subsequently, primers were commercially synthesised with the forward primer of each pair labelled with one of four fluorescent dyes compatible with the 3500xL Genetic Analyser (G5 dye set, Life Technologies, Thermo Fisher), leaving LIZ as the label of the commercially prepared size standard ladder. Primer sequences are listed in Table 1.

**Table 1:** STR loci and primer sequences for genotyping WSSV

Locus	Forward primer seq 5′-3′	5′primer tail	Reverse seq 5′-3′	Allele size range*
wsv1	TTCCATTTCTTCTCCACTATC	PET	TGGAGAAGGTTTGTTACCTC	171-228
wsv2	GCGAGACAGAGAAGACTAAG	6-FAM	TCATCGTTTTGAATTGTGGC	362-389
wsv3	ATTTCTATGAGGATGGTTACG	VIC	CGTCTTCACAATCAATAACAC	146-164
wsv4	GTTTTACTGTTGGGCACTAC	6-FAM	CATACAAGCTCCAGTTCCAG	162-195
wsv6	GACAACACCCCTCGTACC	6-FAM	TCACTATCTGCATCCTTATTCTC	260-281
wsv7	TTAAGGGACTATAATGGCAAC	6-FAM	GCACCACTGAAATGAATAAAC	374-386
wsv8	AGATGAATCAGACGAATCGG	PET	AGAACAAAGCAACGAAACTG	196-202
wsv10	CTTTACTTTCTTCCATGTTCG	6-FAM	TAAAATTAATCCTCCCTTTCC	86-95
wsv11	CTGTGGTACCTGACTGTAATG	PET	AATATCGGTTTCTTCGTTATC	89-92
wsv12	GGTGATAAAGCGTTTCTGAG	NED	AAATACTGAACTGGCAGAGG	88-94
wsv13	CATAACTTTGATTACGGTTCC	VIC	AACCTCACAAAAGTGTTGAC	85-91
wsv14	TGGTAGCTTTTATCTTCAAGG	NED	TTGTCCGTATCTGATGTTATC	58-71
wsv15	CGCATCTTCTAGTACAGTTG	VIC	CAACACATTCTCCCATTCTTG	247-271
wsv16	GCTGTTGTTCTTGAGTGTTG	6-FAM	AACGACAATGAATTTGATAGC	59-62
wsv17	AAGACAAAAGTGAGTTTGAGG	NED	TAGGTTACAGCCTACCCTTAG	118-148
wsv18	GGATTTATTCAACGGTATTTG	VIC	CATCTGCAATTTCCATTTC	116-136
wsv19	AAGTCTCTACCTCGAATGAAG	NED	TAGAAATACTTCTCCCACCAC	116-125
wsv20	AGAGAGAACATATCCCGTACC	VIC	CTACCTCATTCTCCTCTTCAG	129-150
wsv21	TGGGCGCATTGTTAAATTG	6-FAM	TGAGTGAAGGAGGTAATGATG	286
wsv22	AATTCTCAAGAGAGGAGGAAC	6-FAM	GAAGATGATTGGGATGAGG	62-68
wsv23	GTAATTTGCTGGTTTCTTACG	6-FAM	TTCCATTTGTACACTTCAATG	146-152
wsv24	ATGAAGGGCTGTAGTTGTAG	6-FAM	CACGGAAAATACTAGCGTTG	271-310
wsv25	ATCTCCTTCTAGCTCGGC	NED	GTTTGAAGTTGTTGGAGAGC	275-281
wsv26	TCAACGACGAGATTGTAGAG	6-FAM	TGAAGGATCGTAAACAACCC	182-197
wsv27	CTACTAGCAGATACCGGAAG	6-FAM	GGTCGTTTTCTTCATACACG	132-141
wsv28	ATAACGAGCCTGTTTCTGAG	PET	CGTTTTCCATTAACAGCTCC	250-253
wsv29	GGTAAAATGGGAGTACAGAAG	VIC	TAACAACACCCAATAACAATG	68-74
wsv30	GTGTTGCAGACTCTAAAGACC	VIC	CTCGTAATCAAAATCTTCCAC	263-290
wsv31	ACCCTCAACCAATATTCGTC	NED	AAGCCTTCAGATTTGGTACG	209-224
wsv32	CTTTGAGTCACTACAGCCAG	NED	TTTGGAAGAGTTGTACAGGG	176-185
wsv33	GTTTGAAAAGGTGCGAGTAG	PET	GGGCGTTGAATTAATCGTG	342-354
wsv34	AAGGATGCAGATAGTGACAG	PET	TCTCTTCTGAATCTTGGCAG	151-196
wsv35	GTGGACTCCTGATAGTGTTC	VIC	GGGCTCTACATCACATCATC	281-296
wsv36	GTAGGTTTGAGTTGAGGAGG	6-FAM	TCCAGACAATGAAATGGGAG	112-124

*allele size range according to conditions provided by the 3500xL instrument, POP-7 polymer and 50-mm capillary array. Size shift may be experienced if alternative conditions are used

DNA was extracted, using a DNeasy Blood and Tissue Kit (QIAGEN), from the same prawn used to determine the sequence of WSSV-AU. For preliminary optimisation each STR locus, amplification was performed as a monoplex using 7.5 µL of Multiplex Master Mix (QIAGEN), 2 pmol each of forward and reverse primer, 2.5 µL of DNA, and a volume balance with sterile nuclease-free water to 15 µL. Following initial denaturation at 94 °C for 15 minutes, the reactions were cycled 40 times at 94 °C for 30 seconds, at the estimated annealing temperatures of 54, 57 or 58 °C for 30 seconds, and 72 °C for 1 minute, with a single final extension at 72 °C for 10 minutes. The reaction products were resolved using 1.5% agarose gel electrophoresis. The presence or absence of single amplicons of the expected size and the observed relative intensity were used to optimise amplification of the loci with adjustments to the annealing temperature and the inclusion of Q-solution (QIAGEN) in the mix. These empirical results were used subsequently to fine-tune and optimise multiplexed reactions.

The final optimised method targeted 34 loci in six PCRs with further multiplexing of the amplicons into three reactions prior to resolution. The loci in each PCR are shown in Table 2. PCR mixes consisted of 7.5 µL of Multiplex Master Mix (QIAGEN), 1.5 µL of Q solution (QIAGEN) where used, 2 pmol of each primer, 2.5 µL of DNA, and a volume balance of sterile nuclease-free water to 15 µL per reaction. Following initial denaturation at 94 °C for 15 minutes, the reactions were cycled 40 times at 94 °C for 30 seconds, at the respective annealing temperature (see Table 2) for 45 seconds and 72 °C for 45 seconds, with a single final extension at 72 °C for 10 minutes. Amplicons were diluted 1 in 50 using Milli-Q water and further multiplexed by combining PCRs 1, 2 and 3 (Read1), and PCRs 5 and 6 (Read3). Read2 consisted only of PCR4. Reads 1, 2 and 3 were resolved using fragment analysis by capillary electrophoresis with a 3500xL Genetic Analyser (Life Technologies, Thermo Fisher), with fragment sizes determined by comparison with the labelled size marker (GeneScan 600, Life Technologies, Thermo Fisher) using GeneMarker (Soft Genetics).

**Table 2:** Locus multiplexing and amplification conditions

	PCR1	PCR2	PCR3	PCR4	PCR5	PCR6
Annealing temp. °C	53	57	59	54	56	58
Loci	WSV8	WSV4	WSV3	WSV1	WSV24	WSV2
2 pmol of each forward and reverse primer for each locus per reaction	WSV12	WSV7	WSV16	WSV6	WSV31	WSV17
		WSV13	WSV21	WSV10		WSV20
		WSV15	WSV36	WSV11		WSV22
		WSV32		WSV14		WSV25
				WSV18		WSV26
				WSV19		WSV27
				WSV23		WSV28
				WSV29		WSV33
				WSV30		WSV34
						WSV35
Q-solution **(**1.5 µL per reaction)	Yes	No	No	No	No	No

The robustness of the optimised technique was tested based on consistency in fragment lengths in repeated tests of the same DNA sample, comparison of data from re-extracted DNA from the same sample, and comparison among three operators. The sensitivity was estimated through comparison with Biosecurity Sciences Laboratory’s (BSL) standard diagnostic PCR (optimised from Sritunyalucksana *et al*. [27] to accommodate laboratory conditions).

### Samples from the Australian outbreak

The STR technique was applied to every Australian sample that tested PCR-positive for WSSV at BSL during the outbreak and surveillance in 2016-8, i.e., 462 samples, as listed in Table 3. These comprised samples from each infected farm property and from surveillance samples of the surrounding waterways and bays. High-throughput nucleic acid extraction used a MagMAX Viral Isolation Kit (Thermo Fisher Scientific) on a KingFisher™ Flex 96 magnetic particle processor (Thermo Fisher Scientific). The manufacturer’s instructions were followed, except the sample size was increased to 100 µL of homogenate, and an additional wash was included before elution.

**Table 3:** Sources of white spot syndrome virus DNA from Queensland, Australia

Year	Area/property (letter represent farms in Logan area of Brisbane)	Site/pond*	Sample species	Number	Genotype
2016	A	11	*P. monodon*	10	LG1
	A	13	*P. monodon*	9	LG1
	B	22	*P. monodon*	4	LG1
	C	7	*P. monodon*	20	LG1
	C	14	*P. monodon*	11	LG1
	C	Inlet channel	*P. monodon*	6	LG1
	D	1, 2 & 4	*P. monodon*	28	LG1
	E	19	*P. monodon*	1	LG1
	E	25	*P. monodon*	3	LG1
	A	11	*P. monodon*	10	LG1
2017	E	Inlet channel	*Scylla serrata*	2	LG1
	E	Inlet channel	*P. monodon*	3	LG1
	E	Inlet channel	*Melicertus plebejus*	1	LG1
	E	Inlet channel	*Scylla serrata*	1	LG2
	E	Inlet channel	*P. monodon*	3	LG1
	E	Inlet channel	*P. monodon*	10	LG1
	E	1	*P. monodon*	7	LG5
	E			3	LG1
	E	1	*P. monodon*	10	LG1
	E	1	*P. monodon*	9	LG1
	E	8	*P. monodon*	1	LG1
	E	8	*P. monodon*	10	LG1
	E	10	*P. monodon*	20	LG1
	E	10	*P. monodon*	10	LG1
	E	12	*P. monodon*	7	LG1
	E			3	LG6
	E	15	*P. monodon*	8	LG1
	E			2	LG5
	E	15	*P. monodon*	3	LG1
	E			7	LG5
	E	15	*P. monodon*	10	LG1
	E	18	*P. monodon*	4	LG1
	E	31	*P. monodon*	4	LG3
	E			1	LG7
	E	39	*P. monodon*	10	LG5
	E	39	*P. monodon*	8	LG5
	E	47	*P. monodon*	4	LG3
	E	47	*P. monodon*	6	LG3
	E	47	*P. monodon*	5	LG3
	E	47	*P. monodon*	10	LG3
	E	48	*P. monodon*	3	LG1
	E	49	Sand crab	2	LG1
	E	50	*P. monodon*	2	LG1
	E	50	*P. monodon*	6	LG1
	E			1	LG7
	E	50	*P. monodon*	3	LG1
	E			1	LG4
	E			1	LG5
	E			5	LG7
	E	51	*P. monodon*	5	LG1
	E	51	Sand crab	2	LG1
	E	53	*P. monodon*	8	LG1
	E	55	*P. monodon*	2	LG1
	E		*P. monodon*	2	LG4
	E	56	*P. monodon*	7	LG1
	E	56	*P. monodon*	10	LG1
	E	56	*P. monodon*	2	LG1
	E	59	*P. monodon*	7	LG1
	E	Settlement pond	*P. monodon*	10	LG1
	E	Settlement 2	*P. monodon*	3	LG1
	E	Settlement	*P. monodon*	10	LG1
	E	Settlement 7	*P. monodon*	6	LG1
	E	Settlement 6	*P. monodon*	10	LG1
	E	Outlet drain	*P. monodon*	4	LG1
	E	Outlet drain	*P. monodon*	9	LG1
	E	Outlet drain	*P. monodon*	2	LG1
	G	4	*P. monodon*	9	LG1
				1	LG2
	H	19	*P. monodon*	7	LG1
	H	13	*P. monodon*	1	LG1
				9	LG3
	Logan River**		*Metapenaeus bennettae*	8	LG3
			*P. monodon*	1	LG3
			*P. monodon*	1	LG1
				1	LG3
			*P. monodon*	1	LG1
			*M. bennettae*	3	LG1
2017	Moreton Bay**		*M. bennettae*	2	MB1
			*P. merguiensis*	1	MB1
			*P. esculentus*	5	MB2
			*M. bennettae*	4	MB1
			*Melicertus plebejus*	1	MB1
			*M. bennettae*	6	MB1
			*P. esculentus*	2	MB1
			*M. bennettae*	12	MB1
			*Unknown*	9	MB1
2018	Moreton Bay**		*Thalamita crenata*	9	MB6
			*M. bennettae*	48	MB3
				2	MB4
			*P. esculentus*	1	MB5
				9	MB6
				15	MB8
				1	MB10
				1	MB11
			*P. merguiensis*	2	MB3
				8	MB6
			*T. crenata*	14	MB12
				4	MB7
			*M. bennettae*	1	MB1
				37	MB6
				1	MB9
			*P. esculentus*	11	MB1
			*M. bennettae*	47	MB1
			*M. bennettae*	15	MB1

*Where the same site/pond is listed more than once, these represent different sampling occasions

**Where same species is listed more than once, these represent different sampling locations within the same area

Two frozen prawn tissue samples from the feed causing the 1999 Darwin incident (see Introduction) were also tested. DNA was extracted using a DNeasy Blood and Tissue Kit (QIAGEN).

### Samples of imported crustacean retail material

A total of 245 samples from 46 different imported crustacean-based food products were purchased from local and national chain retail outlets. Products included green prawns and marinated green prawn tails, cooked prawns, processed prawn products (cooked and raw, such as prepared dumplings and similar products), crab meat and crab products. Cooked products were included only to expand on spatial representation of WSSV genotypes, but they were not expected to be a potential direct source of viable virus.

DNA extractions and the WSSV-detection PCRs were conducted by BSL as described above. The test-positive DNA extracts (Table 4) were used for STR genotyping.

**Table 4:** Sources of white spot syndrome virus DNA from outside Australia

Stated source		Year	Sample identity	Route of access	Presentation	Species
China		2016-7	C1-C5	Retail. Supermarket 1 deli counter	Loose green prawn tails	*P. vannamei*
		2016-7	C6-C10	Retail. Supermarket 2 deli counter	Loose green prawn tails	*P. vannamei*
		2016-7	C16-C20	Retail. Supermarket 1 deli counter	Loose green marinaded prawn tails	Unknown
		2016-7	C21-C25	Retail. Pre-packaged, brand 3, supermarket	Frozen green marinaded prawn tails	*P. vannamei*
		2016-7	C26-C30	Retail. Pre-packaged, brand 4, supermarket	Frozen green marinaded prawn tails	*P. vannamei*
		2016-7	C71-75	Retail. Pre-packaged, brand 4, supermarket	Frozen green prawn tails	*P. vannamei*
		2016	IT14, IT44	CSIRO AAHL*	DNA extracted from imported prawns	Unknown
		Unknown	IT2, IT5, IT6, IT9, IT12, IT38	CSIRO AAHL*	DNA extracted from imported prawns	Unknown
Vietnam		2016-7	V11-V15	Retail. Pre-packaged, brand 3, supermarket	Frozen green marinaded prawn tails	*P. vannamei*
		2016-7	V16-V20	Retail. Supermarket 1 deli counter	Loose green marinaded prawn tails	Unknown
		2016-7	V21-V25	Retail. Pre-packaged, brand 3, supermarket	Frozen green marinaded prawn tails	*P. vannamei*
		2016-7	V26-30	Retail. Pre-packaged, brand 13, supermarket	Loose green prawn tails	*P. monodon*
		2016-7	V56-V60	Retail. Pre-packaged, brand 5, supermarket	Frozen breaded green prawn tails	*P. vannamei*
		2016-7	V96-V100	Retail. Pre-packaged, brand 6, supermarket	Frozen crab cake	*Portunus haani*
		2016-7	V76-V80	Retail. Pre-packaged, brand 4, supermarket	Frozen cooked prawn tails	*P. vannamei*
		2016-7	V111-115	Retail. Pre-packaged, brand 11, supermarket	Frozen processed complete menu product	Unknown
		2016-7	V151-155	Retail. Pre-packaged, brand 12, supermarket	Frozen processed complete menu product	Unknown
		2016-7	V156-160	Retail. Pre-packaged, brand 12, supermarket	Frozen processed complete menu product	Unknown
		2016	IT17, IT49, IT50	CSIRO AAHL*	DNA extracted from imported prawns	*P. monodon*
		Unknown	IT22, IT24	CSIRO AAHL*	DNA extracted from imported prawns	*P. monodon*
		2016	IT18	CSIRO AAHL*	DNA extracted from imported prawns	*P. vannamei*
		Unknown	IT23	CSIRO AAHL*	DNA extracted from imported prawns	*P. vannamei*
		2016	IT21, IT25, IT40-43, IT46-48	CSIRO AAHL*	DNA extracted from imported prawns	Unknown
		Unknown	IT20, IT27-37, IT39	CSIRO AAHL*	DNA extracted from imported prawns	Unknown
		2013	IT45	CSIRO AAHL*	DNA extracted from imported prawns	Unknown
Thailand		2016-7	T1-T5	Retail. Pre-packaged, brand 7, supermarket	Frozen cooked prawn tails	Unknown
		2016-7	T6-T10	Retail. Supermarket 1 deli counter	Loose cooked prawn tails	*P. vannamei*
		2016-7	T16-T20	Retail. Pre-packaged, brand 8	Dried prawn tails	Unknown
		2016-7	T41-T45	Retail. Supermarket 1 deli counter	Loose cooked prawn tails	*P. vannamei*
		2016-7	T101-T105	Retail. Pre-packaged, brand 4, supermarket	Frozen processed complete menu product	*P. vannamei*
		2016-7	T106-T110	Retail. Pre-packaged, brand 10, supermarket	Frozen processed complete menu product	*P. vannamei*
		2016-7	T116-120	Retail. Pre-packaged, brand 3, supermarket	Frozen processed complete menu product	*P. vannamei*
		2018	Thai2	Supplier name withheld	Prawns in ethanol	
		1998	C-98	Dr. A Dhar	DNA in ethanol	*P. monodon*
		2017	F-17	Dr. A Dhar	DNA in ethanol	Dried feed
Malaysia		Unknown	IT1, IT3-4, IT7-8, IT10-11, IT13, IT15, IT19, IT26	CSIRO AAHL*	DNA extracted from imported prawns	Unknown
Indonesia		2016-7	I86-I90	Retail. Pre-packaged, brand 9, supermarket	Frozen cooked crab meat	*Portunus pelagicus*
		1999	D1	Dr. A Dhar	DNA in ethanol	*P. monodon*
	Sengkang, S. Sulawesi	2018	Sul_A1-Sul_A12;	Dr. M. Rimmer	Pleiopods in ethanol	*P. monodon*
	Takalar, S. Sulawesi	2018	Sul_B1-Sul_B3	Dr. M. Rimmer	Pleiopods in ethanol	*P. monodon*
India	Tamil Nadu	‘O’: period 2002-2004 ‘N’: period 2014-2017	OTN1, OTN2, OTN3, NTN1, NTN2, NTN3, NTN4	Dr. S. Hameed	DNA on FTA cards	‘O’ *P. monodon* ‘N’ *P. vannamei*
	Andhra Pradesh		OAP1, NAP1, NAP2, NAP3	Dr. S. Hameed	DNA on FTA cards	
				Dr. S. Hameed	DNA on FTA cards	
	Kerala		OKE1, NKE1, NKE2, NKE3, NKE4, NKE5, NKE6, NKE7			
				Dr. S. Hameed	DNA on FTA cards	
	Odisha		OOD1	Dr. S. Hameed	DNA on FTA cards	
	West Bengal		OWB1, NWB1	Dr. S. Hameed	DNA on FTA cards	
	Gujarat		OGU1	Dr. S. Hameed	DNA on FTA cards	
Kingdom of Saudi Arabia		2011	SA1-2	Dr V. Alday Sanz	Prawns in ethanol	*P. indicus*
Iran	Khuzestan	2018	IR1-IR7	Dr. M. Afsharnasab	Prawns in ethanol	*P. vannamei*
	Sistan and Baluchestan	2018	IR8-IR15	Dr. M. Afsharnasab	Prawn tissue in ethanol	*P. vannamei*
Ecuador		2018	E1-E6	Supplier name withheld	Prawns in ethanol	*P. vannamei*
USA	Arizona retail	1996	A-96	Dr. A Dhar	DNA in ethanol	
	South Carolina mariculture	1997	B1	Dr. A Dhar	DNA in ethanol	
			B2	Dr. A Dhar	DNA in ethanol	
			B3	Dr. A Dhar	DNA in ethanol	
	South Carolina retail	1997	B4	Dr. A Dhar	DNA in ethanol	
Honduras		1999	D2-99	Dr. A Dhar	DNA in ethanol	Unknown
		2002	E-02	Dr. A Dhar	DNA in ethanol	*P. vannamei*

*WSSV detected during testing as part of the importation process. Testing conducted by CSIRO Australian Animal Health Laboratories, Geelong, VIC

### Samples of penaeid material from other regions of the world

Samples from other global regions were provided either as ethanol-preserved tissue, DNA in ethanol or DNA fixed on FTA cards (GE Healthcare, Biostrategy, VIC). Prior to STR genotyping, DNA extractions from tissue and detection of WSSV by PCR were conducted by BSL as described above, or DNA was extracted using a DNeasy Blood and Tissue Kit, and tested similarly for the presence of WSSV DNA. FTA cards were processed according to the manufacturer’s instructions. The WSSV-positive DNA extracts or FTA cards (Table 4) were used for STR genotyping.

### Comparison of STR genotyping resolution sensitivity with other loci

One sample of each of the STR genotypes identified from the affected farms in Logan and from Moreton Bay were tested by PCR and amplicon sequencing of ORFs 75, 94 and 125 [19] as described previously [18].

### Data analysis

Basic analysis of data such as allele frequency and Nei’s genetic identity was done using Genalex v6.4 [28] with *a priori* assumptions of WSSV origin as stated on retail packages or by the donor.

Such analysis may be hindered by prior assumptions of origin and the dichotomous nature of widely used phylogenetic trees that use genetic distance. Hence, the entire dataset of genotypes without prior clustering according to the stated source or origin was used to create a more appropriate minimum spanning tree using the GeoBURST full MST algorithm in PHYLOViZ v2 [29].

## Results

Thirty-six STR markers were identified, including some with perfect tandem repeats and some with imperfect repeats but variation in copy number between reported genome sequences. Testing for robustness showed consistency in fragment lengths among repeated tests of the same DNA extract, comparison of data from re-extracted DNA from the same sample, and comparison among three operators, with 34 markers. Two markers (WSV5 and WSV9) were discarded from the locus panels because they did not work optimally at a shared annealing temperature. The sensitivity of the genotyping was determined to be equivalent to the diagnostic PCR; STR fragments were generated from samples that had diagnostic PCR Ct values as high as 38 when tested by BSL, although the larger fragments were not always observed in samples with Cts above 35. For approximately 20% of the processed retail products, more than two thirds of the loci were not amplified, and where this occurred, even when WSSV detection PCR Cts were less than 35, this was presumably because of DNA degradation as a result of the cooking, drying or other processing.

A total of seven genotypes were observed from samples taken from infected ponds in farms and in the Logan River (LG1 to LG7, Tables 3 and 5), with the majority being of genotype LG1. The seven genotypes differed in only one or two loci. Where samples were taken from the same site or pond on different occasions, and hence tested on different occasions, the results were consistent, which further demonstrates the robustness of the allele calls. A total of twelve genotypes were observed from samples taken from Moreton Bay (MB1 to MB12, Tables 3 and 5). In 2017, two genotypes were apparent. MB1 predominated and only one sample (five individuals) showed MB2. In 2018, all MB types were observed except MB2. There was no common genotype found in both the Logan area and in Moreton Bay, with one locus (WSV24) consistently showing genotypic difference between the two areas.

**Table 5:**
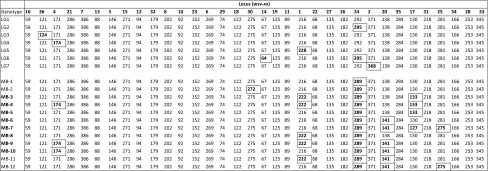
Genotypes observed in samples taken in Queensland 2016-2018. Boxed alleles indicate those that differ from LG1

A large range of alleles was observed from the samples originating from outside Queensland, as indicated by the actual allele size range shown in Table 1, compared to alleles shown for Queensland samples. Most loci were highly polymorphic, while some showed only two or three alleles globally. One locus appeared monomorphic (WSV21) and was retained in the panel as a control marker. Many samples originating from regions where WSSV is endemic showed infection with multiple genotypes, seen as more than one allele at individual loci. Where this occurred, all of the possible genotype iterations were determined, as this approach would not impede subsequent analyses that rely upon allele frequencies and distances. The allelic data are summarised in Table 6 as allele frequencies for *a priori* given global regions. Table 7 shows Nei’s genetic identity between the same *a priori* regions.

**Table 6:** Allele frequencies related to the *a priori*-stated origin of the sample

Locus	Allele	Vietnam	China	Malaysia	Thailand	Indonesia	Saudi Arabia	USA	Honduras	Ecuador	India	Iran	Darwin	QLD
**WSV16**	**59**	0.111	0.994	0.042	0.886	0.718	1.000	0.276	0.500	1.000	1.000	1.000	1.000	1.000
	**62**	0.889	0.006	0.958	0.114	0.282	0.000	0.724	0.500	0.000	0.000	0.000	0.000	0.000
**WSV36**	**112**	0.000	0.000	0.000	0.015	0.000	0.000	0.000	0.000	0.000	0.000	0.000	0.000	0.000
	**115**	0.000	0.026	0.008	0.176	0.000	0.000	0.000	0.000	0.000	0.090	0.000	0.000	0.000
	**118**	1.000	0.800	0.992	0.809	0.385	1.000	0.483	0.000	0.500	0.401	0.200	1.000	0.000
	**121**	0.000	0.174	0.000	0.000	0.615	0.000	0.517	1.000	0.500	0.389	0.800	0.000	0.944
	**124**	0.000	0.000	0.000	0.000	0.000	0.000	0.000	0.000	0.000	0.120	0.000	0.000	0.056
**WSV4**	**162**	0.000	0.052	0.407	0.000	0.000	0.000	0.000	0.000	0.000	0.383	0.800	0.000	0.000
	**165**	0.228	0.555	0.034	0.393	0.205	0.000	0.000	0.000	0.000	0.018	0.200	0.000	0.000
	**168**	0.184	0.013	0.275	0.000	0.308	0.000	0.017	0.000	0.500	0.138	0.000	0.900	0.000
	**171**	0.059	0.006	0.000	0.094	0.410	1.000	0.983	1.000	0.000	0.054	0.000	0.000	0.778
	**174**	0.295	0.000	0.284	0.255	0.077	0.000	0.000	0.000	0.000	0.000	0.000	0.000	0.222
	**177**	0.203	0.361	0.000	0.243	0.000	0.000	0.000	0.000	0.000	0.006	0.000	0.000	0.000
	**180**	0.012	0.000	0.000	0.000	0.000	0.000	0.000	0.000	0.000	0.000	0.000	0.000	0.000
	**183**	0.000	0.013	0.000	0.015	0.000	0.000	0.000	0.000	0.000	0.000	0.000	0.000	0.000
	**186**	0.019	0.000	0.000	0.000	0.000	0.000	0.000	0.000	0.000	0.000	0.000	0.000	0.000
	**189**	0.000	0.000	0.000	0.000	0.000	0.000	0.000	0.000	0.000	0.048	0.000	0.000	0.000
	**192**	0.000	0.000	0.000	0.000	0.000	0.000	0.000	0.000	0.000	0.180	0.000	0.100	0.000
	**195**	0.000	0.000	0.000	0.000	0.000	0.000	0.000	0.000	0.500	0.174	0.000	0.000	0.000
**WSV21**	**286**	1.000	1.000	1.000	1.000	1.000	1.000	1.000	1.000	1.000	1.000	1.000	1.000	1.000
**WSV7**	**374**	0.000	0.258	0.000	0.000	0.000	0.000	0.000	0.000	0.000	0.000	0.000	0.000	0.000
	**377**	0.145	0.000	0.000	0.000	0.000	0.000	0.000	0.000	0.000	0.000	0.200	0.000	0.000
	**380**	0.001	0.200	0.280	0.000	0.000	0.000	0.000	0.000	1.000	0.844	0.000	1.000	0.000
	**383**	0.854	0.542	0.585	1.000	0.897	1.000	1.000	1.000	0.000	0.156	0.800	0.000	0.000
	**386**	0.000	0.000	0.136	0.000	0.103	0.000	0.000	0.000	0.000	0.000	0.000	0.000	1.000
**WSV13**	**85**	1.000	0.994	0.928	1.000	1.000	0.000	1.000	1.000	0.500	0.940	0.800	0.800	0.000
	**88**	0.000	0.006	0.072	0.000	0.000	1.000	0.000	0.000	0.000	0.006	0.200	0.200	1.000
	**91**	0.000	0.000	0.000	0.000	0.000	0.000	0.000	0.000	0.500	0.054	0.000	0.000	0.000
**WSV3**	**146**	0.325	0.948	0.517	0.578	0.000	1.000	0.000	0.000	0.000	0.425	0.000	0.000	1.000
	**149**	0.000	0.000	0.000	0.000	0.000	0.000	0.000	0.000	0.000	0.000	0.000	1.000	0.000
	**155**	0.000	0.000	0.000	0.047	0.000	0.000	0.414	0.500	0.000	0.000	0.000	0.000	0.000
	**161**	0.000	0.052	0.000	0.000	0.000	0.000	0.000	0.000	0.000	0.383	0.457	0.000	0.000
	**164**	0.675	0.000	0.483	0.375	1.000	0.000	0.586	0.500	1.000	0.192	0.543	0.000	0.000
**WSV15**	**247**	0.000	0.000	0.000	0.000	0.077	0.000	0.000	0.000	0.000	0.000	0.000	0.000	0.000
	**259**	0.072	0.000	0.000	0.000	0.000	0.000	0.000	0.000	0.000	0.000	0.000	0.000	0.000
	**265**	0.928	1.000	1.000	1.000	0.923	1.000	1.000	1.000	1.000	1.000	0.200	1.000	0.000
	**271**	0.000	0.000	0.000	0.000	0.000	0.000	0.000	0.000	0.000	0.000	0.800	0.000	1.000
**WSV12**	**88**	0.002	0.000	0.000	0.109	0.000	0.000	0.000	0.000	0.000	0.000	0.000	0.000	0.000
	**91**	0.925	0.974	0.657	0.892	1.000	0.000	0.862	1.000	1.000	1.000	1.000	1.000	0.000
	**94**	0.073	0.026	0.343	0.000	0.000	1.000	0.138	0.000	0.000	0.000	0.000	0.000	1.000
**WSV32**	**176**	0.010	0.000	0.000	0.000	0.000	0.000	0.000	0.000	0.000	0.000	0.000	0.000	0.000
	**179**	0.915	1.000	0.657	1.000	0.000	1.000	0.000	0.500	1.000	1.000	0.314	0.600	1.000
	**182**	0.003	0.000	0.343	0.000	1.000	0.000	1.000	0.500	0.000	0.000	0.686	0.400	0.000
	**185**	0.072	0.000	0.000	0.000	0.000	0.000	0.000	0.000	0.000	0.000	0.000	0.000	0.000
**WSV8**	**196**	0.344	0.000	0.000	0.006	0.000	0.000	0.000	0.000	0.000	0.000	0.000	0.000	0.000
	**199**	0.616	1.000	0.314	0.994	1.000	1.000	1.000	1.000	1.000	1.000	1.000	1.000	0.000
	**202**	0.040	0.000	0.686	0.000	0.000	0.000	0.000	0.000	0.000	0.000	0.000	0.000	1.000
**WSV10**	**86**	0.000	0.000	0.000	0.000	0.000	0.000	0.000	0.000	0.000	0.000	0.229	0.000	0.000
	**89**	0.994	0.232	0.415	0.349	1.000	0.000	0.586	1.000	1.000	0.934	0.771	1.000	0.000
	**92**	0.005	0.626	0.576	0.651	0.000	1.000	0.414	0.000	0.000	0.066	0.000	0.000	1.000
	**95**	0.001	0.142	0.008	0.000	0.000	0.000	0.000	0.000	0.000	0.000	0.000	0.000	0.000
**WSV23**	**146**	0.378	0.000	0.000	0.000	0.000	0.000	0.000	0.000	0.000	0.000	0.000	0.000	0.000
	**149**	0.354	0.839	0.572	0.892	0.795	1.000	0.017	1.000	1.000	1.000	1.000	1.000	0.000
	**152**	0.268	0.161	0.428	0.109	0.205	0.000	0.983	0.000	0.000	0.000	0.000	0.000	1.000
**WSV6**	**260**	0.018	0.000	0.000	0.000	0.000	0.000	0.000	0.000	0.000	0.000	0.800	0.000	0.000
	**263**	0.092	0.000	0.407	0.000	0.000	0.000	0.000	0.000	0.000	0.006	0.200	0.100	0.000
	**266**	0.459	0.684	0.576	0.563	0.795	0.000	0.414	0.500	1.000	0.940	0.000	0.500	0.000
	**269**	0.367	0.000	0.008	0.328	0.000	1.000	0.000	0.000	0.000	0.054	0.000	0.000	1.000
	**272**	0.002	0.316	0.008	0.000	0.205	0.000	0.586	0.500	0.000	0.000	0.000	0.000	0.000
	**275**	0.062	0.000	0.000	0.000	0.000	0.000	0.000	0.000	0.000	0.000	0.000	0.000	0.000
	**278**	0.000	0.000	0.000	0.109	0.000	0.000	0.000	0.000	0.000	0.000	0.000	0.000	0.000
	**281**	0.000	0.000	0.000	0.000	0.000	0.000	0.000	0.000	0.000	0.000	0.000	0.400	0.000
**WSV29**	**68**	0.049	0.000	0.000	0.106	0.000	0.000	0.000	0.000	0.000	0.078	0.400	0.000	0.000
	**71**	0.886	0.200	0.288	0.642	0.923	1.000	0.586	1.000	0.000	0.084	0.600	1.000	0.000
	**74**	0.065	0.800	0.712	0.252	0.077	0.000	0.414	0.000	1.000	0.838	0.000	0.000	1.000
**WSV18**	**116**	0.049	0.000	0.000	0.132	0.205	0.000	0.000	0.000	0.000	0.000	0.800	0.000	0.000
	**119**	0.718	1.000	1.000	0.868	0.795	1.000	1.000	1.000	1.000	1.000	0.200	0.200	0.000
	**122**	0.157	0.000	0.000	0.000	0.000	0.000	0.000	0.000	0.000	0.000	0.000	0.000	1.000
	**125**	0.076	0.000	0.000	0.000	0.000	0.000	0.000	0.000	0.000	0.000	0.000	0.000	0.000
	**136**	0.000	0.000	0.000	0.000	0.000	0.000	0.000	0.000	0.000	0.000	0.000	0.800	0.000
**WSV30**	**263**	0.000	0.103	0.000	0.100	0.000	0.000	0.000	0.000	0.000	0.000	0.200	0.500	0.000
	**266**	0.011	0.000	0.004	0.188	0.205	0.000	0.000	0.000	0.000	0.641	0.000	0.400	0.000
	**269**	0.014	0.129	0.000	0.000	0.000	1.000	0.000	0.000	0.000	0.054	0.000	0.100	0.000
	**272**	0.137	0.445	0.576	0.267	0.410	0.000	1.000	1.000	1.000	0.090	0.800	0.000	0.056
	**275**	0.480	0.097	0.407	0.199	0.308	0.000	0.000	0.000	0.000	0.024	0.000	0.000	0.944
	**278**	0.165	0.226	0.008	0.000	0.000	0.000	0.000	0.000	0.000	0.192	0.000	0.000	0.000
	**281**	0.150	0.000	0.004	0.018	0.000	0.000	0.000	0.000	0.000	0.000	0.000	0.000	0.000
	**284**	0.041	0.000	0.000	0.000	0.077	0.000	0.000	0.000	0.000	0.000	0.000	0.000	0.000
	**287**	0.001	0.000	0.000	0.229	0.000	0.000	0.000	0.000	0.000	0.000	0.000	0.000	0.000
	**290**	0.002	0.000	0.000	0.000	0.000	0.000	0.000	0.000	0.000	0.000	0.000	0.000	0.000
**WSV14**	**58**	0.145	0.013	0.000	0.000	0.000	0.000	0.000	0.000	0.000	0.018	0.114	0.000	0.000
	**61**	0.803	0.065	0.966	0.208	0.795	0.000	1.000	1.000	0.500	0.078	0.886	1.000	0.000
	**64**	0.052	0.813	0.025	0.792	0.205	0.000	0.000	0.000	0.500	0.257	0.000	0.000	0.056
	**67**	0.000	0.103	0.008	0.000	0.000	1.000	0.000	0.000	0.000	0.647	0.000	0.000	0.944
	**71**	0.000	0.006	0.000	0.000	0.000	0.000	0.000	0.000	0.000	0.000	0.000	0.000	0.000
**WSV19**	**116**	0.006	0.077	0.000	0.000	0.000	0.000	0.000	0.000	0.000	0.000	0.800	0.000	0.000
	**119**	0.419	0.923	1.000	0.892	1.000	1.000	0.966	0.500	1.000	1.000	0.200	1.000	0.000
	**122**	0.537	0.000	0.000	0.109	0.000	0.000	0.000	0.500	0.000	0.000	0.000	0.000	0.000
	**125**	0.038	0.000	0.000	0.000	0.000	0.000	0.034	0.000	0.000	0.000	0.000	0.000	1.000
**WSV11**	**89**	0.006	0.065	0.000	0.109	0.000	1.000	0.000	0.000	1.000	0.000	0.000	0.000	1.000
	**92**	0.994	0.935	1.000	0.892	1.000	0.000	1.000	1.000	0.000	1.000	1.000	1.000	0.000
**WSV1**	**171**	0.000	0.000	0.000	0.000	0.615	0.000	0.000	0.000	0.000	0.000	0.000	0.000	0.000
	**174**	0.001	0.000	0.000	0.346	0.000	0.000	0.000	0.000	0.000	0.000	0.000	0.000	0.000
	**186**	0.003	0.000	0.000	0.000	0.000	0.000	0.000	0.000	0.000	0.000	0.000	0.000	0.000
	**192**	0.144	0.000	0.000	0.000	0.000	0.000	0.000	0.000	0.000	0.006	0.000	0.000	0.000
	**195**	0.001	0.000	0.000	0.000	0.000	0.000	0.000	0.000	0.000	0.000	0.000	0.000	0.000
	**198**	0.270	0.413	0.068	0.258	0.385	0.000	0.000	0.500	0.500	0.186	0.000	0.000	0.000
	**201**	0.000	0.148	0.004	0.000	0.000	0.000	0.000	0.000	0.000	0.000	0.000	0.000	0.000
	**204**	0.001	0.090	0.000	0.302	0.000	0.000	0.000	0.500	0.500	0.808	0.000	0.000	0.000
	**210**	0.001	0.000	0.000	0.000	0.000	0.000	0.000	0.000	0.000	0.000	0.000	0.000	0.000
	**216**	0.542	0.348	0.924	0.094	0.000	1.000	1.000	0.000	0.000	0.000	1.000	1.000	0.722
	**222**	0.038	0.000	0.004	0.000	0.000	0.000	0.000	0.000	0.000	0.000	0.000	0.000	0.222
	**228**	0.000	0.000	0.000	0.000	0.000	0.000	0.000	0.000	0.000	0.000	0.000	0.000	0.056
**WSV22**	**62**	0.000	0.019	0.004	0.000	0.615	0.000	0.000	0.000	0.000	0.024	0.000	0.000	0.000
	**65**	1.000	0.981	0.852	1.000	0.385	0.000	1.000	1.000	1.000	0.976	1.000	1.000	0.000
	**68**	0.000	0.000	0.144	0.000	0.000	1.000	0.000	0.000	0.000	0.000	0.000	0.000	1.000
**WSV27**	**132**	0.019	0.168	0.021	0.000	0.000	0.000	0.000	0.000	0.000	0.000	0.000	0.000	0.000
	**135**	0.500	0.006	0.000	0.094	0.000	0.000	0.000	0.000	0.000	0.000	0.000	0.000	1.000
	**138**	0.204	0.826	0.979	0.906	1.000	1.000	1.000	1.000	1.000	1.000	1.000	1.000	0.000
	**141**	0.277	0.000	0.000	0.000	0.000	0.000	0.000	0.000	0.000	0.000	0.000	0.000	0.000
**WSV26**	**182**	0.066	0.310	0.720	0.299	0.000	1.000	0.345	0.000	0.000	0.150	0.000	0.000	1.000
	**188**	0.014	0.000	0.000	0.000	0.000	0.000	0.000	0.000	0.000	0.000	0.800	0.000	0.000
	**191**	0.046	0.000	0.000	0.047	0.000	0.000	0.000	0.000	0.000	0.000	0.000	0.000	0.000
	**197**	0.874	0.690	0.280	0.654	1.000	0.000	0.655	1.000	1.000	0.850	0.200	1.000	0.000
**WSV24**	**271**	0.009	0.000	0.017	0.000	0.000	0.000	0.000	0.000	0.000	0.030	0.000	0.000	0.000
	**274**	0.019	0.000	0.551	0.000	0.308	0.000	0.000	0.000	0.000	0.048	0.000	0.000	0.000
	**277**	0.454	0.116	0.004	0.050	0.615	0.000	1.000	1.000	0.000	0.006	0.800	0.000	0.000
	**280**	0.336	0.310	0.004	0.094	0.000	0.000	0.000	0.000	0.000	0.012	0.200	0.600	0.000
	**283**	0.022	0.129	0.000	0.299	0.000	0.000	0.000	0.000	1.000	0.012	0.000	0.400	0.000
	**286**	0.148	0.142	0.008	0.144	0.077	1.000	0.000	0.000	0.000	0.006	0.000	0.000	0.000
	**289**	0.000	0.271	0.000	0.000	0.000	0.000	0.000	0.000	0.000	0.054	0.000	0.000	0.611
	**292**	0.002	0.032	0.136	0.135	0.000	0.000	0.000	0.000	0.000	0.389	0.000	0.000	0.278
	**295**	0.010	0.000	0.000	0.196	0.000	0.000	0.000	0.000	0.000	0.443	0.000	0.000	0.111
	**298**	0.000	0.000	0.000	0.009	0.000	0.000	0.000	0.000	0.000	0.000	0.000	0.000	0.000
	**301**	0.000	0.000	0.280	0.000	0.000	0.000	0.000	0.000	0.000	0.000	0.000	0.000	0.000
	**304**	0.000	0.000	0.000	0.050	0.000	0.000	0.000	0.000	0.000	0.000	0.000	0.000	0.000
	**310**	0.000	0.000	0.000	0.023	0.000	0.000	0.000	0.000	0.000	0.000	0.000	0.000	0.000
**WSV2**	**362**	0.001	0.000	0.000	0.000	0.000	0.000	0.000	0.000	0.000	0.000	0.000	0.000	0.000
	**365**	0.005	0.006	0.000	0.000	0.000	0.000	0.000	0.000	0.000	0.000	0.000	0.000	0.000
	**368**	0.000	0.000	0.542	0.191	0.000	0.000	0.000	0.000	0.000	0.000	0.000	0.000	0.056
	**371**	0.245	0.439	0.008	0.070	0.000	0.000	0.897	0.000	0.000	0.647	0.000	0.000	0.944
	**374**	0.508	0.555	0.441	0.739	1.000	1.000	0.103	1.000	0.000	0.323	0.200	1.000	0.000
	**377**	0.242	0.000	0.008	0.000	0.000	0.000	0.000	0.000	0.000	0.000	0.000	0.000	0.000
	**380**	0.001	0.000	0.000	0.000	0.000	0.000	0.000	0.000	0.000	0.006	0.800	0.000	0.000
	**383**	0.000	0.000	0.000	0.000	0.000	0.000	0.000	0.000	0.000	0.024	0.000	0.000	0.000
	**389**	0.000	0.000	0.000	0.000	0.000	0.000	0.000	0.000	1.000	0.000	0.000	0.000	0.000
**WSV20**	**129**	0.000	0.000	0.000	0.185	0.000	0.000	0.000	0.000	0.000	0.000	0.000	0.000	0.000
	**132**	0.000	0.000	0.000	0.000	0.308	0.000	0.000	0.000	0.000	0.000	0.000	0.000	0.000
	**135**	0.225	0.000	0.004	0.094	0.000	0.000	0.000	0.500	0.000	0.000	1.000	1.000	0.000
	**138**	0.624	0.974	0.860	0.721	0.615	1.000	1.000	0.500	1.000	1.000	0.000	0.000	0.722
	**141**	0.147	0.026	0.136	0.000	0.000	0.000	0.000	0.000	0.000	0.000	0.000	0.000	0.278
	**144**	0.005	0.000	0.000	0.000	0.000	0.000	0.000	0.000	0.000	0.000	0.000	0.000	0.000
	**150**	0.000	0.000	0.000	0.000	0.077	0.000	0.000	0.000	0.000	0.000	0.000	0.000	0.000
**WSV35**	**281**	0.014	0.058	0.000	0.000	0.205	0.000	0.448	0.500	0.000	0.006	0.800	0.000	0.000
	**284**	0.985	0.942	1.000	0.853	0.718	1.000	0.552	0.500	1.000	0.994	0.200	1.000	1.000
	**287**	0.001	0.000	0.000	0.144	0.077	0.000	0.000	0.000	0.000	0.000	0.000	0.000	0.000
	**290**	0.000	0.000	0.000	0.003	0.000	0.000	0.000	0.000	0.000	0.000	0.000	0.000	0.000
	**296**	0.001	0.000	0.000	0.000	0.000	0.000	0.000	0.000	0.000	0.000	0.000	0.000	0.000
**WSV17**	**118**	0.000	0.000	0.000	0.000	0.000	0.000	0.000	0.000	0.000	0.269	0.000	0.000	0.000
	**121**	0.005	0.103	0.000	0.000	0.000	0.000	0.000	0.000	0.000	0.000	0.000	0.000	0.000
	**124**	0.015	0.245	0.004	0.000	0.000	0.000	0.000	0.000	0.000	0.000	0.000	0.000	0.000
	**127**	0.547	0.103	0.136	0.006	0.000	0.000	0.000	0.000	0.000	0.431	0.000	0.000	0.056
	**130**	0.237	0.465	0.847	0.578	0.923	1.000	1.000	1.000	0.000	0.096	0.600	1.000	0.778
	**133**	0.110	0.084	0.004	0.135	0.077	0.000	0.000	0.000	0.000	0.192	0.000	0.000	0.167
	**136**	0.011	0.000	0.008	0.047	0.000	0.000	0.000	0.000	0.000	0.000	0.114	0.000	0.000
	**139**	0.075	0.000	0.000	0.094	0.000	0.000	0.000	0.000	1.000	0.000	0.000	0.000	0.000
	**142**	0.000	0.000	0.000	0.032	0.000	0.000	0.000	0.000	0.000	0.012	0.000	0.000	0.000
	**145**	0.000	0.000	0.000	0.094	0.000	0.000	0.000	0.000	0.000	0.000	0.000	0.000	0.000
	**148**	0.000	0.000	0.000	0.015	0.000	0.000	0.000	0.000	0.000	0.000	0.286	0.000	0.000
**WSV31**	**209**	0.000	0.000	0.000	0.000	0.000	0.000	0.000	0.000	0.000	0.000	0.400	0.000	0.000
	**212**	0.001	0.000	0.000	0.000	0.000	0.000	0.000	0.000	0.000	0.000	0.000	0.000	0.000
	**215**	0.000	0.000	0.000	0.000	0.000	0.000	0.000	0.000	0.000	0.006	0.000	0.000	0.000
	**218**	0.688	1.000	0.996	0.953	1.000	1.000	0.724	1.000	1.000	0.802	0.600	1.000	1.000
	**221**	0.001	0.000	0.004	0.047	0.000	0.000	0.000	0.000	0.000	0.192	0.000	0.000	0.000
	**224**	0.311	0.000	0.000	0.000	0.000	0.000	0.276	0.000	0.000	0.000	0.000	0.000	0.000
**WSV25**	**275**	0.001	0.000	0.000	0.000	0.000	0.000	0.000	0.000	0.000	0.000	0.000	0.000	0.111
	**278**	0.863	0.813	0.856	0.938	1.000	0.000	1.000	1.000	1.000	0.988	1.000	1.000	0.000
	**281**	0.136	0.187	0.144	0.062	0.000	1.000	0.000	0.000	0.000	0.012	0.000	0.000	0.889
**WSV34**	**151**	0.000	0.000	0.000	0.000	0.077	0.000	0.000	0.000	0.000	0.000	0.000	0.000	0.000
	**154**	0.000	0.000	0.000	0.000	0.000	0.000	0.000	0.000	0.000	0.000	0.000	0.400	0.000
	**157**	0.000	0.103	0.000	0.000	0.000	0.000	0.000	0.000	0.000	0.024	0.000	0.000	0.000
	**160**	0.000	0.000	0.000	0.000	0.308	0.000	0.000	0.000	0.000	0.000	0.000	0.000	0.000
	**163**	0.010	0.000	0.000	0.006	0.615	0.000	0.724	1.000	0.000	0.078	0.800	0.400	0.000
	**166**	0.542	0.110	0.288	0.109	0.000	0.000	0.276	0.000	0.000	0.054	0.200	0.000	1.000
	**169**	0.328	0.181	0.000	0.340	0.000	0.000	0.000	0.000	0.000	0.192	0.000	0.000	0.000
	**172**	0.018	0.077	0.000	0.000	0.000	0.000	0.000	0.000	0.000	0.006	0.000	0.000	0.000
	**175**	0.075	0.213	0.000	0.000	0.000	1.000	0.000	0.000	0.000	0.000	0.000	0.200	0.000
	**178**	0.001	0.000	0.004	0.179	0.000	0.000	0.000	0.000	0.000	0.006	0.000	0.000	0.000
	**181**	0.007	0.026	0.000	0.243	0.000	0.000	0.000	0.000	0.000	0.000	0.000	0.000	0.000
	**184**	0.000	0.000	0.144	0.000	0.000	0.000	0.000	0.000	0.000	0.000	0.000	0.000	0.000
	**187**	0.000	0.000	0.564	0.000	0.000	0.000	0.000	0.000	1.000	0.641	0.000	0.000	0.000
	**190**	0.020	0.174	0.000	0.029	0.000	0.000	0.000	0.000	0.000	0.000	0.000	0.000	0.000
	**193**	0.000	0.000	0.000	0.070	0.000	0.000	0.000	0.000	0.000	0.000	0.000	0.000	0.000
	**196**	0.000	0.116	0.000	0.023	0.000	0.000	0.000	0.000	0.000	0.000	0.000	0.000	0.000
**WSV28**	**250**	1.000	1.000	1.000	1.000	1.000	1.000	1.000	1.000	1.000	1.000	1.000	1.000	0.000
	**253**	0.000	0.000	0.000	0.000	0.000	0.000	0.000	0.000	0.000	0.000	0.000	0.000	1.000
**WSV33**	**342**	0.000	0.000	0.000	0.000	0.205	0.000	0.000	0.000	0.000	0.000	0.000	0.000	0.000
	**345**	0.000	0.006	0.000	0.000	0.000	0.000	0.000	0.500	0.000	0.072	0.000	0.000	1.000
	**348**	0.683	0.987	1.000	0.935	0.718	1.000	1.000	0.500	1.000	0.904	1.000	1.000	0.000
	**351**	0.317	0.006	0.000	0.065	0.077	0.000	0.000	0.000	0.000	0.024	0.000	0.000	0.000
	**354**	0.001	0.000	0.000	0.000	0.000	0.000	0.000	0.000	0.000	0.000	0.000	0.000	0.000

**Table 7:** Genetic identity between WSSV isolates from different *a priori*-stated sources

Vietnam	China	Malaysia	Thailand	Indonesia	Saudi Arabia	USA	Honduras	Ecuador	India	Iran	Darwin	Queensland	
1.000													Vietnam
0.778	1.000												China
0.808	0.812	1.000											Malaysia
0.829	0.936	0.818	1.000										Thailand
0.777	0.760	0.758	0.816	1.000									Indonesia
0.529	0.661	0.619	0.672	0.530	1.000								Saudi Arabia
0.760	0.740	0.803	0.740	0.834	0.530	1.000							USA
0.762	0.724	0.702	0.777	0.892	0.505	0.848	1.000						Honduras
0.643	0.759	0.688	0.748	0.686	0.474	0.616	0.656	1.000					Ecuador
0.732	0.871	0.755	0.845	0.743	0.541	0.677	0.712	0.821	1.000				India
0.625	0.610	0.609	0.644	0.713	0.410	0.703	0.748	0.532	0.590	1.000			Iran
0.730	0.731	0.718	0.760	0.793	0.538	0.666	0.723	0.664	0.731	0.683	1.000		Darwin
0.311	0.378	0.394	0.326	0.217	0.547	0.317	0.228	0.264	0.319	0.219	0.204	1.000	Queensland

**Fig. 1:**
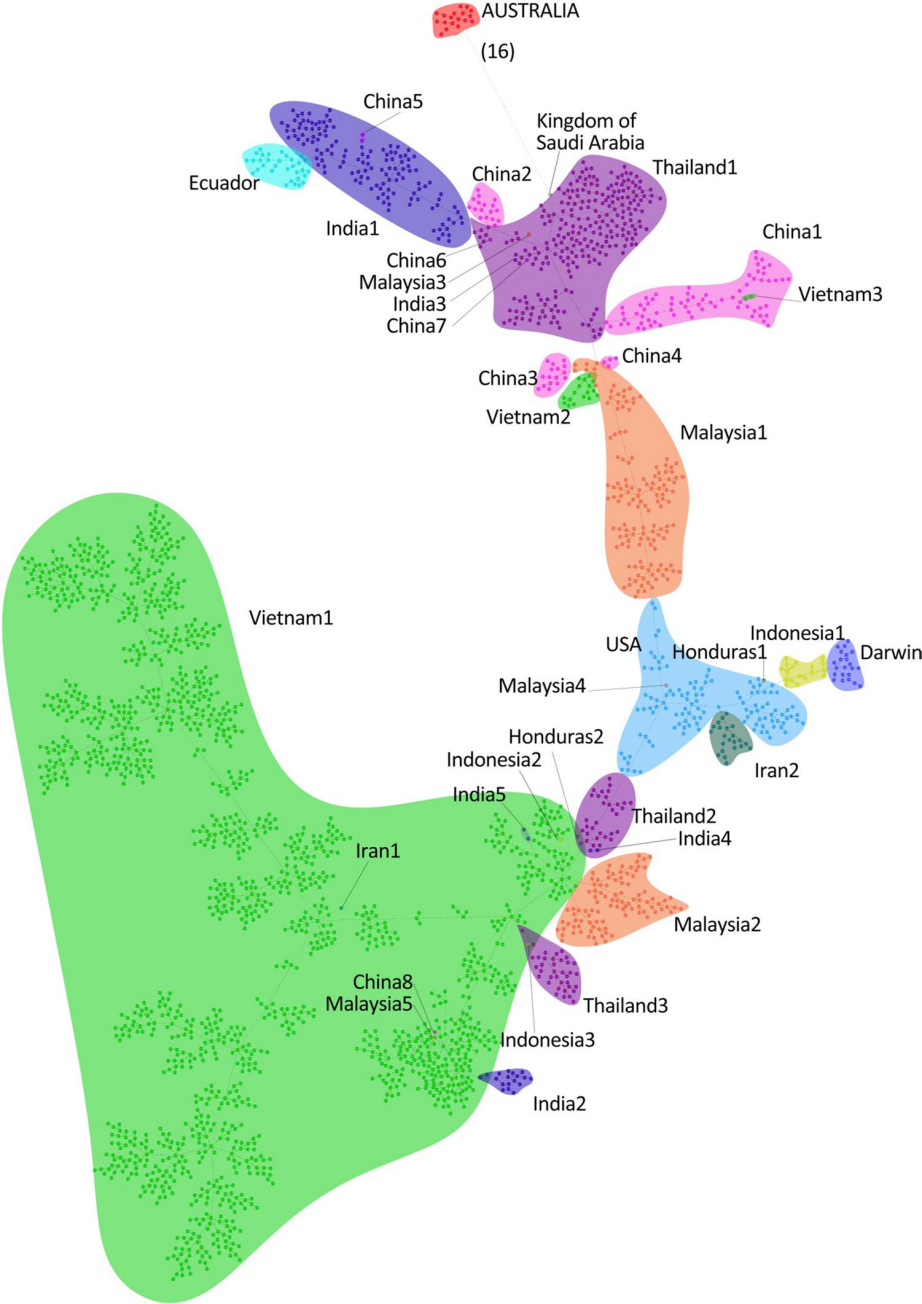
Minimum spanning tree of WSSV genotypes (stylised for ease of labelling and navigation). Where there are multiple clusters from the same region, the numerical codes relate to the following samples: **China1**: C1, C2, C4, C5, C6, C7, C10, C16, C19, C71, C72, C73, C74, C75, IT14. **China2:** IT5. **China3:** IT2. **China4:** IT9. **China5:** IT6, IT12. **China6:** IT38. **China7:** IT44. **China8:** C30. **India1:** NAP1, NAP2, NAP3, NTN3, NTN4, NTN5, NWB1, NKE1, NKE2, NKE3, NKE4, NKE5. **India2:** OTN1, OAP1, OGU1, OWB1. **India3:** NTN2. **India4:** OOD1, OKE1. **India5:** NTN1. **Thailand1:** T16, T19, T20, T101, T103, T105, T106, T108, T110, T116, T119, T120, T121, T122, T123, T124, T125. **Thailand2:** ThC-98. **Thailand3:** ThaiMB, F17, T140, T142, T143. **Malaysia1:** IT7, IT8, IT10. **Malaysia2:** IT1, IT11, IT19. **Malaysia3:** IT4. **Malaysia4:** IT13. **Malaysia5:** IT3. **Malaysia6:** IT15. **Indonesia1:** D1-99, I187, I186. **Indonesia2:** SulA. **Indonesia3:** SulB. **Vietnam1:** IT16, IT17, IT18, IT20, IT21, IT22, IT23, IT25, IT27, IT28, IT29, IT30, IT31, IT32, IT33, IT34, IT35, IT36, IT37, IT39, IT40, IT41, IT42, IT43, IT45, IT46, IT47, IT48, IT49, IT50, V20, V16, V17, V19, V21, V26, V27, V28, V29, V30, V76, V100, V111, V112, V114, V157, V159, V160. **Vietnam2:** IT24. **Vietnam3:** V23. **Honduras1:** HE02. **Honduras2:** H-D2. **Iran1:** IR1, IR2, IR3, IR4, IR5, IR6, IR7. **Iran2:** IR8, IR9, IR10, IR11, IR12, IR13, IR14, IR15

A minimum spanning tree (MST) was created using all genotypes as nodes with no prior assumptions pertaining to the source of the sample, although each genotype node was assigned a colour according to the reported source. Each genotype was represented in the tree only once, so where multiple samples had the same genotype, the node was labelled with only one of them. Multiple samples with the same genotype/node are listed in Table 8. The minimum spanning tree stylised to show the reported source by colour is shown in Figure 1. Relative branch lengths are not depicted in the tree, most of the genotypes (n = 2,516) have a single step of difference to the next node (hereafter termed as level 1), and low numbers of links have levels 2 to 11 (Table 9). There is only one instance of a level exceeding this: the Australian genotype MB1 has 16 levels in the link to Saudi Arabia. At such a high distance and with the jump from 11 to 16 links, the confidence of this suggested link is questionable.

**Table 8:** Samples with identical genotypes. Samples with identical genotypes are not represented in Figure 1. Each genotype/node is represented only once. Labels are as detailed in Table 4

Retained label	Identical genotypes
V16	V18
V76	V77, V79, V80
C16-2	C17, C18, C19-1, C20-1, C21, C22, C23, C24, C25-1
T16	T17, T18, T20-1
T106	T107, T108, T109, T110
T116	T118, T118, T119, T120
T143	T145
I186	I187-1, I188-2, I190-2
I187-2	I188-1, I189
SulA1	SulA2 to SulA12
SulB1	SulB2, SulB3
IR1	IR2 to IR7

**Table 9:** Distribution of linkage levels between nodes in Figure 1

Linkage level	Frequency
1	2516
2	13
3	15
4	18
5	26
6	20
7	8
8	3
9	2
10	3
11	3
12	0
13	0
14	0
15	0
16	1

### Comparison of STR genotyping resolution sensitivity with other loci

The previously identified markers ORFs 75, 94 and 125 [19] were amplified and sequenced from DNA extracted from one of each of the samples with the 19 genotypes identified in SE Queensland. When compared to WSSV-AU [18], which was assigned to genotype LG1, all of these genotypes likewise showed the identical deletion of ORF94 and partial deletion of ORF 75. However, some differences were observed in the ORF125 locus, with several STR genotypes being co-represented by single ORF125 alleles as shown in Table 10. For example, using the ORF125 VNTR, all of the genotypes from the Logan area were identical (5 + 2 partial repeats), yet the STR method identified seven genotypes LG1 to LG7, with LG2 to LG7 showing one or two loci with different alleles to LG1 (Table 10).

**Table 10:** Comparison of STR genotype with three commonly used genotyping loci (for reference, alleles differing from LG1 are marked in **bold**)

STR genotype	STR fragment sizes at loci variable within SE Queensland										ORF 75	ORF94	ORF125 (number of repeats)
	Wsv36	Wsv4	Wsv30	Wsv14	Wsv1	Wsv24	Wsv2	Wsv20	Wsv17	Wsv25			
LG1	121	171	275	67	216	292	371	138	130	281	Deleted	Deleted	5 + 2 partial
LG2	121	171	275	67	216	**295**	371	138	130	281	Deleted	Deleted	
LG3	**124**	171	275	67	216	292	371	138	130	281	Deleted	Deleted	
LG4	121	**174**	275	67	216	292	371	138	130	281	Deleted	Deleted	
LG5	121	171	275	67	**228**	292	371	138	130	281	Deleted	Deleted	
LG6	121	171	275	**64**	216	**295**	371	138	130	281	Deleted	Deleted	
LG7	121	171	275	67	216	292	**368**	138	130	281	Deleted	Deleted	
MB1	121	171	275	67	216	**289**	371	138	130	281	Deleted	Deleted	4 + 1 partial
MB2	121	171	**272**	67	216	**289**	371	138	**133**	281	Deleted	Deleted	Not tested
MB3	121	171	275	67	**222**	**289**	371	138	**133**	281	Deleted	Deleted	10 + 1 partial
MB4	121	**174**	275	67	**222**	**289**	371	138	**133**	281	Deleted	Deleted	
MB5	121	171	275	67	216	**289**	371	138	**133**	281	Deleted	Deleted	
MB11	121	171	275	67	**222**	**289**	371	**141**	**133**	281	Deleted	Deleted	
MB6	121	171	275	67	216	**289**	371	**141**	130	281	Deleted	Deleted	7 + 1 partial
MB8	121	171	275	67	**222**	**289**	371	138	**133**	281	Deleted	Deleted	
MB13	121	171	275	67	216	**289**	371	**141**	**133**	281	Deleted	Deleted	
MB9	121	**174**	275	67	**222**	**289**	371	**141**	**133**	281	Deleted	Deleted	6 + 1 partial
MB10	121	171	275	67	**222**	**289**	371	**141**	130	281	Deleted	Deleted	
MB12	121	171	275	67	216	**289**	371	**141**	130	**275**	Deleted	Deleted	Not tested

## Discussion

This is the first report of the global distribution of WSSV genotypes. Moreover, the samples were tested using a novel genotyping technique applying STRs. This method showed reproducible results when the same sample was retested on different occasions by different operators and when multiple samples were collected from the same pond on different occasions and tested independently.

The STR method showed higher sensitivity to strain differences than previously reported markers. Of the commonly used VNTR markers [19], ORF 94 is deleted in the Australian strains, ORF75 is partially deleted, and it was observed that several STR genotypes could be co-represented by a single ORF125 allele. The results demonstrated that 17 STR genotypes were represented by five ORF125 types, and only one ORF125 allele corresponded to a single STR type.

We believe this is a superior typing method, perhaps even when compared to whole-genome sequencing, as it has been reported that the WSSV genome has been decreasing in size over the years due to loss of selected and possibly redundant genes, particularly envelope-associated protein genes that may have been involved in ancestral host recognition [18, 30]. In particular, when comparing genomes from strains over a temporal range, such large significant deletions can result in elevated *identities in state* between contemporary strains that have undergone the loss of the same redundant regions even though the remaining genomic sequence may have significant mutations, SNPs, and STR differences that demonstrate a lack of relatedness, or *identity by descent* [18]. The STRs reported here are not located within regions observed to be deleted in recently sampled WSSV isolates and therefore are a more appropriate comparative multi-locus tool.

### Global overview

There is a reported history of substantial trade in live aquatic animals, inevitably resulting in transboundary spread of disease [31]. WSSV most likely reached the Americas through importation of *P. monodon* from Asia ([32–36] and discussed below) and rapidly became established in American native species such as *P. vannamei*. Many of the contemporary samples originating from East Asia in this study were *P. vannamei*, which was introduced from the Americas to China on a commercial stock basis in the late 1990s, and to Thailand in 1998, and Indonesia in 2000. *P. vannamei* was subsequently introduced into the aquaculture industries in Vietnam and Malaysia in 2000, and India in 2001, mostly as a result of disease problems (including white spot disease) with the previously predominant farming of *P. monodon* [35, Dr. V. Alday Sanz, personal observation].

The common practice of translocating unscreened or inadequately tested stocks has led to the spread of WSSV back to Asia from the Americas, where WSSV may often be present at low levels in apparently healthy animals, escaping detection, and may be activated subsequently by stressful conditions of transportation or culture [31]. Additionally, the possible movement of infected marine crustaceans through ballast water may be a source of the pathogen as millions of tons of water are moved with little control across the world [37]. It is no surprise, therefore, to observe that the MST in Figure 1 has a mainstream of clusters from the Americas and from Asian sources that are closely linked to each other, forming a “backbone” of related clusters with regional variation forming local clusters among source regions.

Multiple infections by different strains were frequently noted in samples from endemic regions. Reports of similar observations using the larger VNTRs reported by Marks *et al*. [19] have been made previously [38, 39]. Hoa *et al.* [38] reported a correlation of mixed genotype with non-outbreak occasions (defined as < 50% death), while single genotypes were associated with outbreak occasions (100% death). Indeed, coinfection of single animals was not observed in the Australian samples, although some ponds were the source of several genotypes (Table 3). Similarly, in the Khuzestan province of Iran, a single genotype was recovered from an area where WSSV is noted to be highly virulent compared to Sistan and Baluchestan Province, where the disease is manageable (Dr. M. Afsharnasab, personal observation) and from where multiple strains were detected from single samples. Conversely, Walker *et al.* [39] reported multiple infections with strains in diseased and non-diseased prawns, and in the current study, recent strains from India have been recovered from coinfection but showed increased virulence compared to older strains (Dr. S. Hameed, personal observation). Hence, disease expression might be related not only to virus genotype or number of genotypes but also to environmental triggers, the development of tolerance to persistent viral infection in prawns [40], and to immune priming invoked through prior exposure to viral components [41, 42].

### East Asia (Vietnam, China, Thailand, Malaysia)

It was observed that samples from these East Asian regions commonly contained multiple strains of WSSV (seen as multiple alleles in multiple STR loci). These may be *bona fide* examples of coinfection by multiple strains as noted by others [38] or may be a result of cross-contamination in the large processing plants prior to exportation.

In Figure 1, the genotypes observed in samples imported from the main exporters of prawns to Australia (Vietnam, Thailand, China and Malaysia) formed multiple regional clusters that were closely linked to each other, suggesting that the contemporary WSSV strains are largely regional. This may be the result of increased movement regulations [35] and the subsequent formation of localised clusters. The majority of strains from China formed one cluster (China1 in Figure 1), and multiple samples showed identical genotypes or genotypes located in the same cluster. The Chinese strains showed much less diversity than strains from Thailand, Malaysia or Vietnam. However, Figure 1 shows that there also were instances where small pockets and individual sample genotypes reportedly from one East Asian region were located within a larger cluster from a different region. These results almost certainly reflect the transboundary movement of large numbers of broodstock and larvae [32, 36, 43–46]. Alternatively, because the sources of the retail products are stated only as listed on the packaging, there exists the possibility of error, or of the country where the packaging was done differing from the actual source country. Moreover, there have been media reports of alleged smuggling between some of these countries [47, 48] and the importation of prawns from one region to another for further export [49], which would undoubtedly result in small pockets of WSSV genotypes appearing within different regions.

### Indonesia

Several samples from Indonesia collected over a period of almost 20 years showed WSSV genotypes that clustered together – some from *P. monodon, circa* 1999, and some from retail frozen crab meat (*Portunus pelagicus*) purchased in Brisbane, Queensland, in 2017. The location within Indonesia from which these samples originated is unknown.

Fifteen samples of *P. monodon* from two locations on the island of Sulawesi in 2018 were tested. Within each location, all of the samples showed a single genotype, but there were substantial differences between the two sites. The 10 samples labelled “SulA1” originated from Sengkang, an inland lake in the middle of the island, and the single genotype found in all these samples clustered closely with genotypes in a mixed cluster dominated by strains from Vietnam (Vietnam1 in Figure 1). The five samples labelled “SulB1” originated from Takalar on the southwest coast of the Island, on the Makassar Strait. The single genotype found in all these samples clustered closely with genotypes from Thailand (Thailand3 in Figure 1). In both sites, the prawns were separately descended from broodstock imported from Pacific American stocks (Dr. M. Rimmer, personal communication).

### Americas

WSSV was first reported in the Americas in 1995 when a prawn farm in Texas was likely affected by waste from a nearby prawn-processing plant importing product from Asia [32]. Additionally, *P. monodon* was introduced into the USA and Latin America from Asia during the 1980s and 1990s [35] and may have served as another potential source of WSSV, as the disease spread rapidly through Asian countries during the latter part of this time. In 1997, WSSV was reported also in wild prawns in South Carolina [32], some of which are included in this study. The appearance of WSSV in the USA initiated a number of studies of the role of imported retail product as a source of local infection, and it was considered likely that the incursion into the USA could also be attributed to a few related strains having spread from the Asian “epicentre” through importation of frozen product and/or through transport of live animals from Asia [32–34, 36]. In the current study, Figure 1 shows that the WSSV genotypes observed in the USA samples from 1996-7 are linked closely to those in the main producing regions of Asia.

The high prevalence of disease in *P. monodon* stocks in Asia caused a major shift in production to *P. vannamei*, which was imported from the Americas and is native to the west coast of the Americas from Mexico to Peru. Trade in *P. vannamei* from the Americas to Asia continues at a high rate [35]. Accordingly, translocation of broodstock is known to have led to the spread of disease from the Americas back to Asia [31]. In Figure 1, the close links between the Americas and Asia is shown between the genotypes observed from these two continents. Moreover, the STR genotype from one of the earliest (1999) WSSV reports from Honduras, Central America, is located within the USA cluster, suggesting that there were at least some virus transfer events from the USA to Central America.

The genotypes obtained from samples sourced from Ecuador were separated in Figure 1 from the other samples sourced from the Americas and linked only with a cluster formed from newer WSSV strains from India. Interestingly, Flegel and Fegan [13] cite evidence of WSSV in diseased wild Ecuadorian *P. vannamei* from 1996, three years before the reported clinical disease often attributed in the literature to the spread from USA.

### India

White spot disease in India was first noted in 1994 on the east coast, and the following year on the west coast [31], and it then affected the industry in the whole of India. Similar to the eastern Asian countries, the Indian prawn industry transformed from farming *P. monodon* to culturing *P. vannamei* as a result of disease problems with *P. monodon. P. vannamei* was introduced in 2001 from Taiwan [35], but not on a large commercial scale until *circa* 2009 [50]. Sivakumar *et al.* (2018) [51] compared WSSV sequences in Indian prawns from both prior to and after the large-scale introduction of *P. vannamei* and the subsequent disease in *P. vannamei*. They found substantial differences between the two time periods and also the two host species, with the later viruses showing large deletions compared to the earlier viruses. Major deletions of redundant genes have also been noted in other regions in recent years [18, 30, 52, 54], and the deletion sites reported for the newer Indian strains were among those reported for WSSV-AU [18, 51].

A selection of the samples from the Indian study by Sivakumar *et al.* (2018) [51], representing the different provinces of India over the two time periods, were included in the current study (Table 4). The STR genotyping showed a clear demarcation between the two time periods, but not between the provinces. The majority of genotypes from the older samples (prior to 2005) from both coasts formed a cluster (India2) linked to Vietnam1 (Fig. 1), and a smaller cluster (India4) also representing both coasts (Odisha in the east and Kerala in the west) was linked to Thailand1. However, the majority of the genotypes from the new samples (post-2014) formed a separate cluster (India1) with substantial distance from the older samples but with close links to the genotypes from *P. vannamei* sourced from Ecuador, and to the clusters Thailand1 (predominated by *P. vannamei* hosts) and China2 (a small cluster of genotypes obtained from one sample of unknown species). Interestingly, the emergence of these new strains coincided with the importation of *P. vannamei* broodstock from Ecuador (Dr. S. Hameed, personal observation). Two of the newer samples (NTN2 and NTN1 in Table 4, or India3 and India5 in Figure 1) from Tamil Nadu province clustered within Vietnam2 and Thailand1 (Fig. 1).

Despite the similarities in deletions at sites previously reported only for the newer Indian strains and for WSSV-AU, the STR typing showed no evidence of close links between these sample groups, further suggesting that these major deletions are not indicative markers of contemporary strain differentiation, as discussed above.

It was noted also that the samples with the newer strains of WSSV had substantially higher levels of multiple infections with different strains than the older samples. Additionally, the newer strains showed increased virulence compared to the earlier strains from *P. monodon* (Dr. S. Hameed, personal observation).

### Kingdom of Saudi Arabia

The sample from Kingdom of Saudi Arabia (SA1) was sourced from a WSSV incursion and outbreak in 2010-11. Tang *et al*. [52, 53] reported this to be a similar strain to that associated with the incursion into Mozambique and Madagascar in 2012, and it could have originated from the Red Sea, although this was not supported by any genetic evidence apart from the previously unreported deletion of the ORF94 VNTR region not being observed in reports from Asian countries. In the current study it was observed that the SA1 genotype indeed appeared to have no close genotypic link with those sampled from Asia or America using the STR genotyping. Figure 1 shows the closest genotype to be based upon 11 STR differences to a genotype from Thailand, and this is not a persuasive link.

It is interesting to note that the genotype observed in the sample from Saudi Arabia had no discernible link with the genotypes observed from the Persian Gulf or the Gulf of Oman. While most of the prawn mariculture in Saudi Arabia is on the Red Sea coast, it might have been expected that if the source of the 2010-11 incursion was some regional variant of WSSV from the Red Sea, then related variants may be located in the relatively close-by Persian Gulf and Gulf of Oman, which also lead into the Arabian Sea.

### Iran

Seven samples were received from Khuzestan Province, in the northernmost part of the Persian Gulf, where WSSV is noted to be particularly virulent (M. Afsharnasab, personal observation). All seven showed the same single genotype. In Figure 1, this genotype (IR1) aligns with a cluster dominated by Vietnam and also containing genotypes obtained from samples sourced from Malaysia, India, China and Sulawesi. As noted with the Sulawesi samples, the *P. vannamei* samples from Khuzestan are reported to be descendants of imported Pacific American broodstock. It is not known if other samples in the Vietnam cluster may have originated from Pacific America also.

Eight further samples were received from Chabehar, Sistan and Baluchestan Province, on the coast of the Gulf of Oman. In contrast to the samples from Khuzestan, these contained multiple strains, all of which differed from the strain in Khuzestan. The strains observed from Chabehar clustered closest to strains from South Carolina, USA, in 1997, albeit with a level 10 link.

### Australia

Samples of the prawns used as feed associated with the unsustained infection of crustaceans in Darwin Harbour in 1999 were tested and compared to the Queensland strains. The prawns from the Darwin incident showed multiple strains in a similar manner to samples of infected prawns from endemic regions, and no genotype observed was similar to any of the Queensland genotypes. In Figure 1, it can be seen that the Darwin samples align closely to strains from Indonesia in 1999, which confirms previous indications that these prawns were, in fact, imported from Indonesia in 1999 before being inadvertently used as feed in the Darwin research facility.

All of the Queensland genotypes from the Logan farms and Moreton Bay formed a discrete cluster that showed no apparent linkage to other regions represented in Figure 1. The closest genotype to the Australian cluster is the incursion that occurred in Saudi Arabia, but this is a level 16 link and, in addition to the lack of any evidence for a physical epidemiological link, is unlikely to reflect true relatedness.

All PCR-positive samples contained single genotypes, in contrast to the multiple infections noted above in samples from WSSV endemic regions. The rapid progression of disease with a single viral strain per animal is in accordance with the observations of Hoa *et al.* [38] as discussed above, although some ponds in some Queensland farms were the source of several genotypes, but no coinfection was observed (Table 3). Farms A to D had LG1 exclusively, while farm E had all seven LG genotypes and farms G and H had LG1 plus a low frequency of some of the others noted in Farm E. It is unknown at present why farm E had a higher variation of strains. Whether this is a consequence of the large numbers of samples received from this property or whether it is a true reflection of the strain distribution requires further investigation.

The prawns from the Logan farms and river were infected with different genotypes from the prawns sampled from Moreton Bay, with no common strain observed from both areas. However, the strains from the Logan area and Moreton Bay clustered closer together than to those of the other area, forming a single cluster when compared to strains from other regions of the world. The strains from both areas evidently were closely related. Spread from one area to the other with concurrent mutations would be expected to result in the presence of the non-mutated strain as well as mutated ones, so this is unlikely. If the WSSV in SE Queensland was a recent incursion, this raises the possibility that there might have been at least two introductions, most likely from the same source. Further studies are underway to investigate this possibility.

The risk of introduction of pathogens via imported frozen prawns has long been recognised [32, 33]. Lightner *et al*. [32] have suggested that the likely routes of infection include release of untreated wastes from reprocessing plants, disposal of wastes in landfills, where birds consume the material and subsequently contaminate farms and natural fauna, using imported prawns as food for maintenance of other aquatic species, and the use of imported prawns as bait by sports fishermen in coastal waters. The latter scenario has been widely considered to be the likely cause of the WSSV outbreak in Queensland.

However, although the genotyping described above results in the source of the outbreak being undetermined, it provides no evidence to support the premise that the outbreak was caused by recent importation of green prawns from Asia that were intended for human consumption but instead used as bait. The samples tested here were sourced from retail outlets in the Brisbane area immediately after the outbreak was detected and would likely represent the imported green prawns circulating for sale at the time. The samples represented a wide selection of brands and products, and even included cooked and processed products to increase contemporary WSSV representation by exporting regions. Moreover, the samples included product in which WSSV was detected at the stage of importation clearance testing during the year prior to, and immediately following, the outbreak, that provided additional representation from these countries. Hence, while it cannot be assumed that every genotype of WSSV is represented here, the localised clustering observed in Figure 1 implies that the regions at least appear to be recognisable based on genotype.

One alternative possible explanation of the apparent lack of relatedness of the Australian WSSV cluster to others is a long-term undetected reservoir of WSSV in Australia. Although local populations of virus do become established across the globe (Fig. 1), the source links are still recognisable. In contrast, the Australian strains form a cluster that cannot be assigned to a source. However, the data presented here indicate that the possibility of a dormant “native” lineage in Australia needs to at least be considered when investigating the epidemiology of the incursion(s).

In summary, this STR typing technique confirms much of what has been assumed previously regarding the movement of WSSV from Asia to the Americas and back to Asia, with minor mutations to the genotype along this pathway.

From the results of this study, it was not possible to identify the source of the SE Queensland incursion. However, the method described here is a valuable tool to assist further epidemiological analyses. The STR genotyping concept presented here provides a more sensitive typing mechanism than previously reported markers. Such highly discriminatory strain differentiation is invaluable in epidemiological tracing, not only for the SE Queensland incursion but also other incursions and epidemiological analysis on a global scale. Moreover, the STR genotyping of WSSV has potential for application by regulatory bodies investigating transboundary movement of stock infected with WSSV or regulation of commodity package labelling.
